# Supplementary figures and images for: Phagolysosomes break down the membrane of a non-apoptotic corpse independent of macroautophagy
Source: PLoS One. 2024 Nov 21;19(11):e0306435. doi: 10.1371/journal.pone.0306435 (PMC11581207; doi:10.1371/journal.pone.0306435)

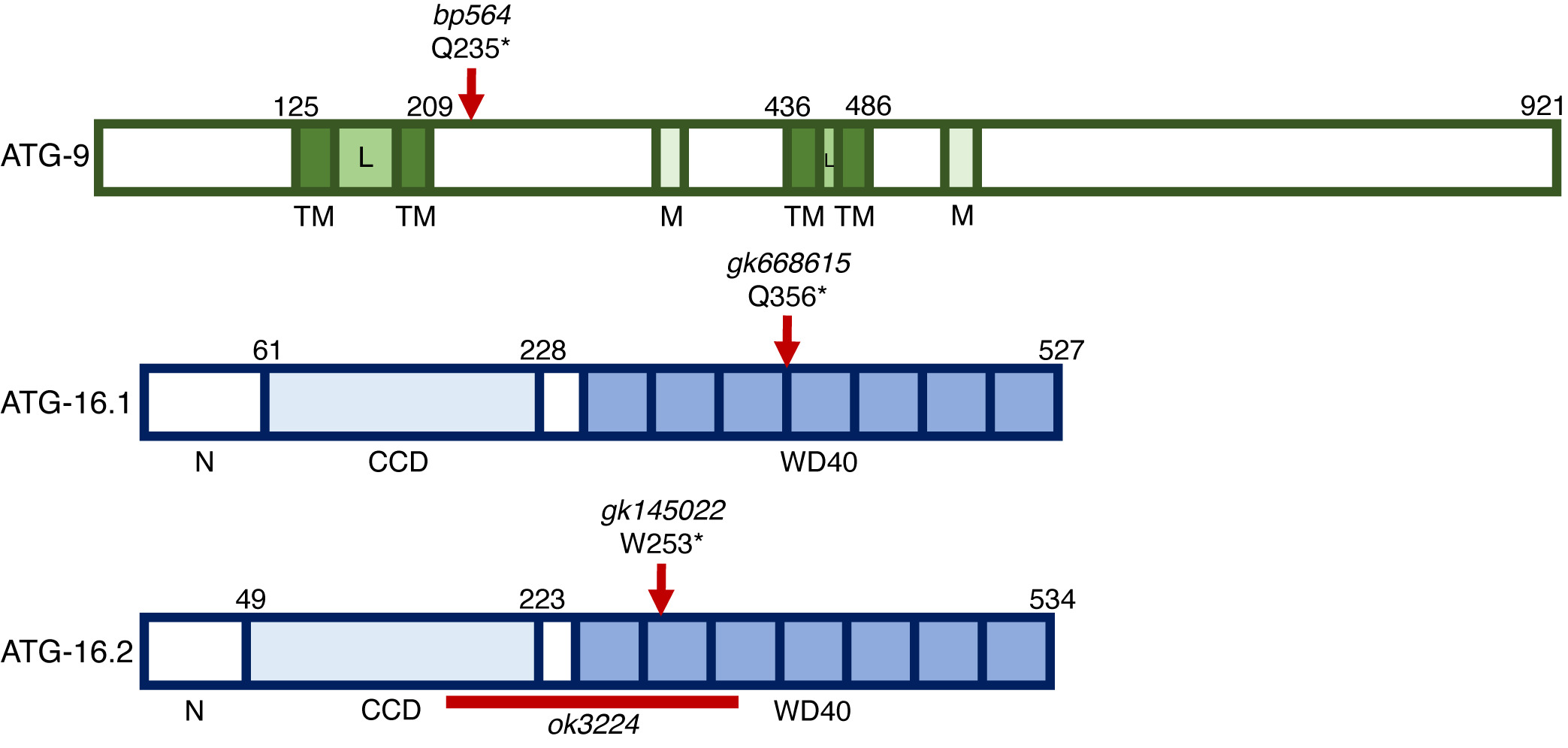

Supplement: S1 Fig — ATG-9 has four transmembrane domains (TM) and two alpha-helices predicted to be partially embedded in the membrane (M), based on the structure of ATG9A [37]. Most of the protein is cytosolic, except for two small luminal domains (L). ATG-16.1 and ATG-16.2 have an N-terminal domain predicted to bind ATG-5 and ATG-12, a central coiled-coil domain (CCD) important for macroautophagy, and a C-terminal WD40 domain important for non-canonical autophagy (LAP/CASM). Positions of point mutations and the ok3224 deletion are indicated. (TIF) [file pone.0306435.s001.tif]

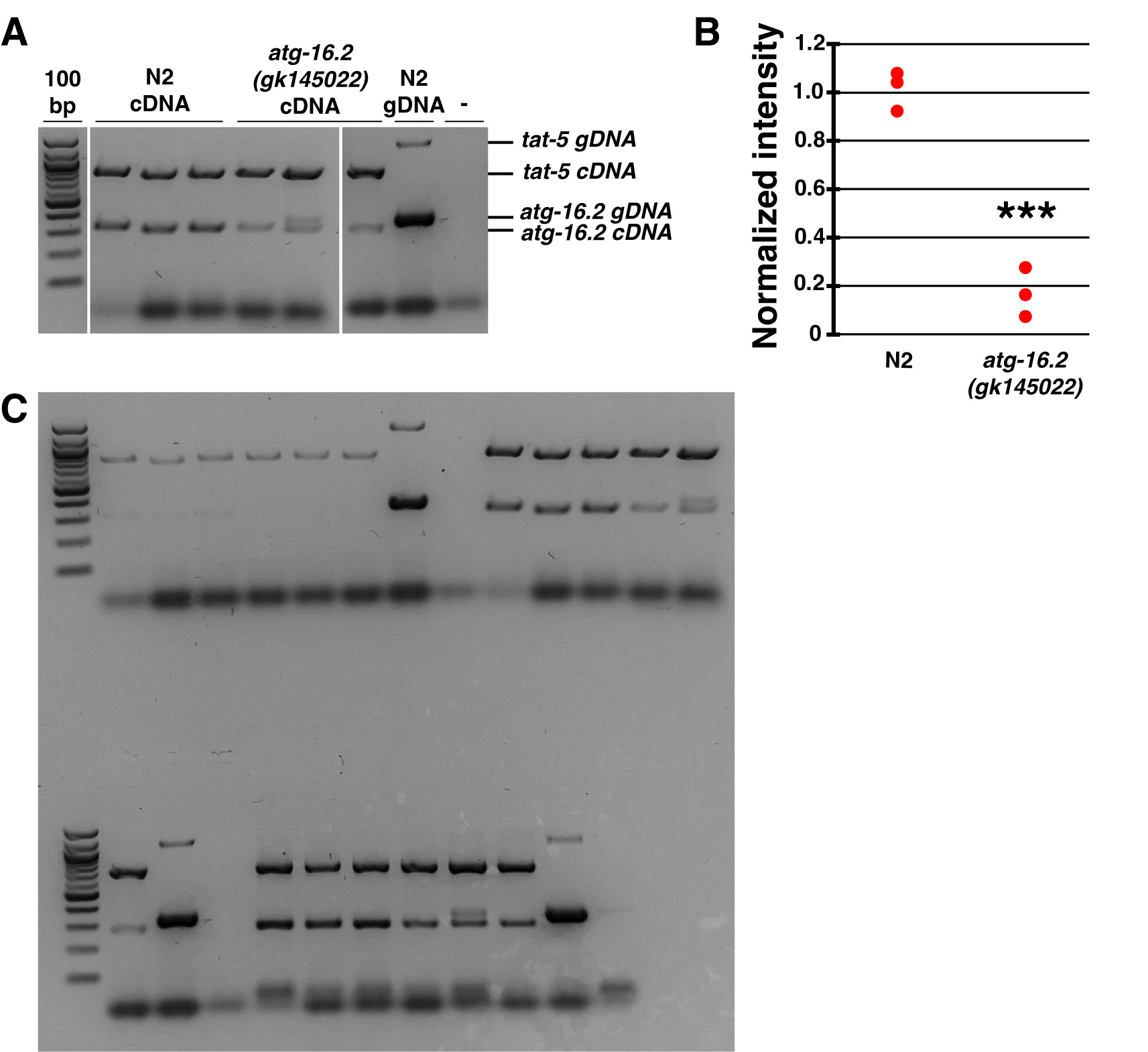

Supplement: S2 Fig — (A) Three biological replicates of wild-type N2 and atg-16.2(gk145022[W253*]) cDNA were co-amplified for atg-16.2 and tat-5 as a loading control. (B) Graph of normalized subtracted fluorescence intensity ratios. The atg-16.2(gk145022[W253*]) mutant band is significantly reduced compared to wild-type using a one-tailed t-test. ***p<0.001. (C) Original gel showing results of 25, 30 (excerpts in panel A), or 35 PCR cycles. (TIF) [file pone.0306435.s002.tif]

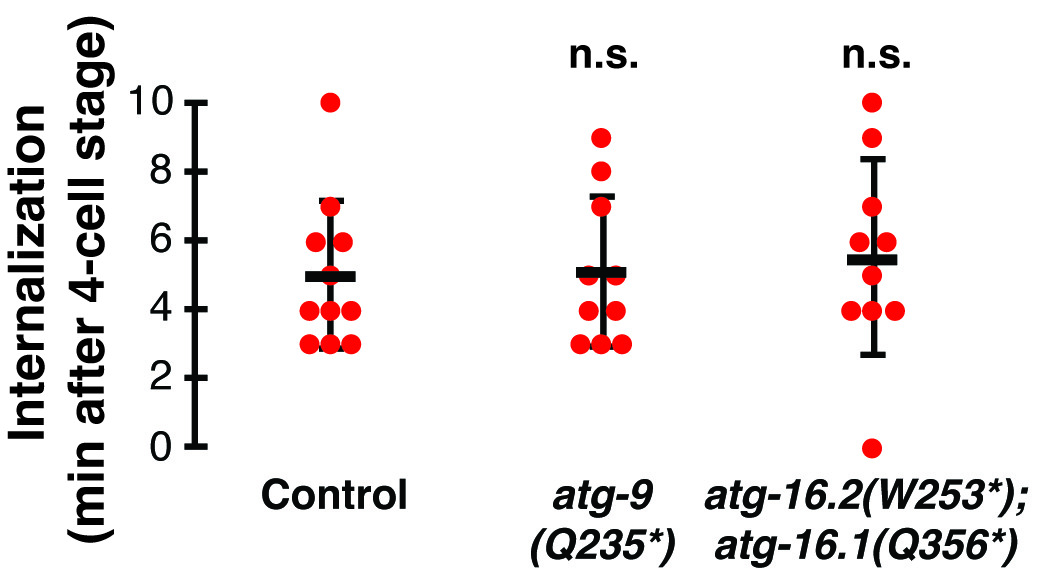

Supplement: S3 Fig — Timing of polar body internalization after the 4-cell stage. Control embryos averaged 5±2 minutes after the 4-cell stage (n = 11). There was no significant delay in internalization in atg-9 single mutants (5±2, n = 10) or atg-16.2(W253*); atg-16.1(Q356*) double mutants (6±3, n = 10). Data are presented as mean ± std dev. One-tailed t-test, p>0.3. (TIF) [file pone.0306435.s003.tif]

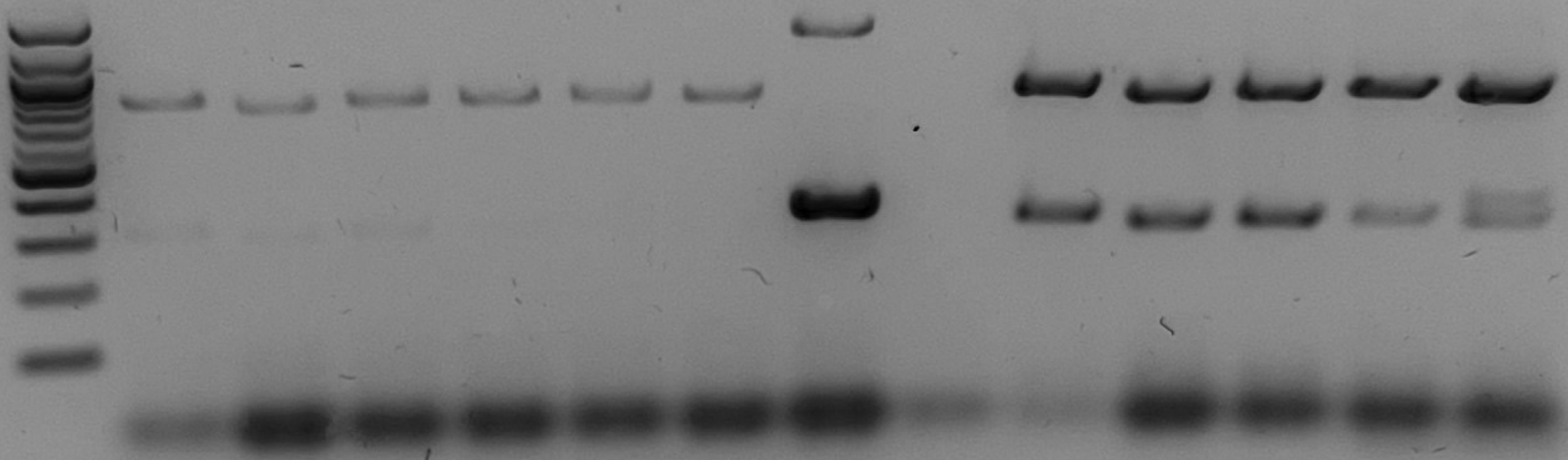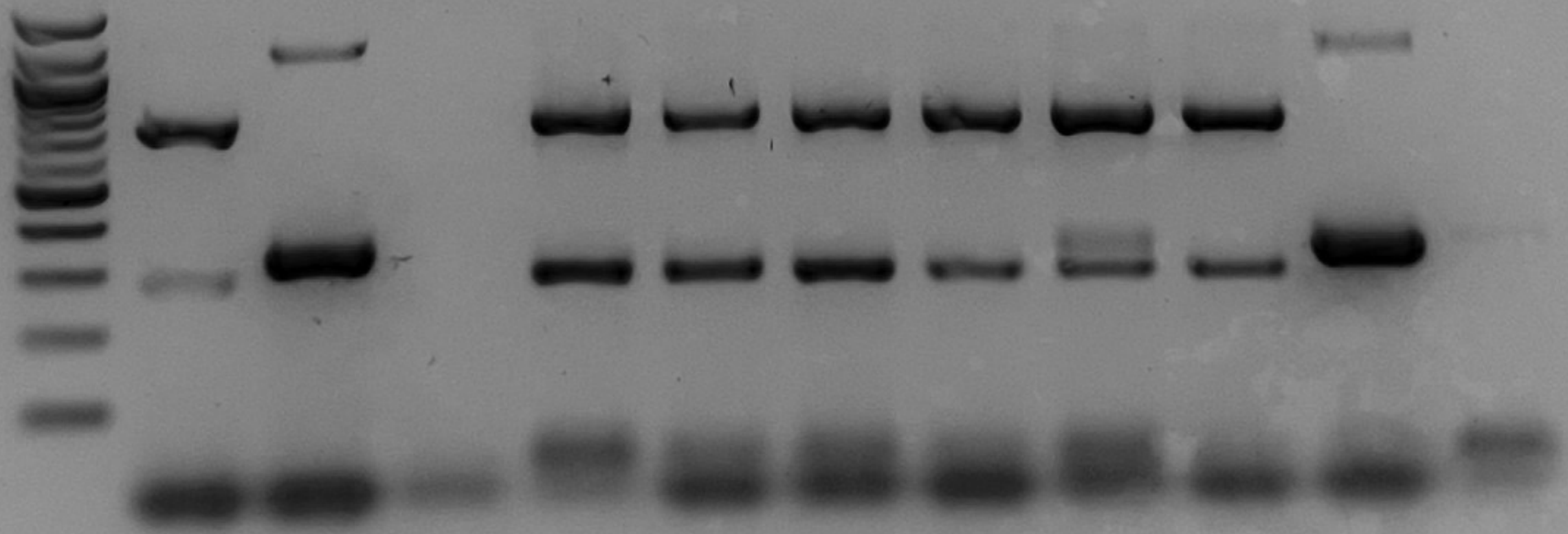

Supplement: S1 Raw image — Original gel showing results of 25, 30 (excerpted in S2A Fig), or 35 PCR cycles after reverse transcription of atg-16.2 mutant mRNA. See also S2C Fig. (PDF) [file pone.0306435.s007.pdf]
