# Supplementary material for: Phagolysosomes break down the membrane of a non-apoptotic corpse independent of macroautophagy
Source: PLoS One. 2024 Nov 21;19(11):e0306435. doi: 10.1371/journal.pone.0306435 (PMC11581207; doi:10.1371/journal.pone.0306435)
Supplement: S1 Table — (PDF) [file pone.0306435.s004.pdf]

S1 Table. Worm strains.

Strain	Genotype	Source
N2	Wild type	[17]
AZ212	*unc-119(ed3) ruIs32[pAZ132: pie-1p::GFP::H2B; unc-119(+)] III*	[38]
COP93	*ttTi5605 II; unc-119(ed3) III*	InVivo Biosystems
FT97	*cdc-42(gk388) / mIn1[dpy-10(e128) mIs14(myo-2::gfp; pes-10::gfp)] xnIs25[cdc-42::GFP::CDC-42; unc-119(+)] II; unc-119(ed3) III*	Jeremy Nance Lab
FT1056	*lgg-1(tm3489) / mIn1[dpy-10(e128) mIs14(myo-2::gfp; pes-10::gfp)] II*	Crossed JJ1610 to RD
HZ1687	*atg-9(bp564) him-5(e1490) V*	[39]
JJ1610	*pkc-3(ok544)/mIn1[dpy-10(e128) mIs14] II; him-8(e1489) IV*	[40]
OD58	*unc-119(ed3) ltIs38[pie-1p::GFP::PH(PLC1delta1), unc-119(+)] III*	[41]
RB2372	*atg-16.2(ok3224) II*	[42]
RD	*lgg-1(tm3489) / dpy-10(e128) unc-4(e120) II*	[43]
VC20426	*atg-16.2(gk145022[W253*]) II and 519 other mutations*	[23]
VC40503	*atg-16.1(gk668615[Q356*]) X and 693 other mutations*	[23]
VIG25	*Is[pVIG57: Pmex-5::mCherry::LGG-2::tbb-2 3'UTR; C.b. unc-119(+)] II; unc-119(ed3) III*	[2]
WEH80	*mIn1[dpy-10(e128) mIs14(myo-2::gfp; pes-10::gfp)] II; ltIs38[pie-1::GFP::PH(PLC1delta1) unc-119(+)] xnIs8[pJN343: nmy-2::NMY-2-mCherry; unc-119(+)] unc-119(ed3) III; lgg-2(tm5755) IV*	Crossed
WEH82	*lgg-1(tm3489) / mIn1[dpy-10(e128) mIs14(myo-2::gfp; pes-10::gfp)] II; ltIs38[pie-1::GFP::PH(PLC1delta1), unc-119(+)] xnIs8[pJN343: nmy-2::NMY-2::mCherry; unc-119(+)] unc-119(ed3) III; lgg-2(tm5755) IV*	Crossed WEH80 to FT1056
WEH95	*xnIs390[pie-1::GFP::ZF1::PH(PLCdelta1), unc-119(+)]; unc-119(ed3) III; Is[mCherry::HistoneH2B] IV*	[44]

WEH224	*Si[pVIG57: Pmex-5::mCherry::LGG-2::tbb-2 3'UTR, C.b. unc-119(+)] II; unc-119(ed3) ruIs32[pAZ132: pie-1::GFP::H2B, unc-119(+)] III*	Crossed VIG25 to AZ212
WEH260	*unc-119(ed3) III; wurIs90[pGF7:pie-1p::mCh::PH::ZF1, unc-119(+)]*	[44]
WEH399	*unc-119(ed3) III; wurIs144[pGF13: pie-1::ZF1::mCherry::his-15, unc-119(+)]*	[19]
WEH464	*wurIs90[pGF7:pie-1::mCh::PH::ZF1, unc-119(+)] II; unc-119(ed3) ltIs38[pie-1::GFP::PH(PLC1delta1), unc-119(+)] III*	Crossed WEH260 to OD58
WEH683	*xnIs390[pie-1::GFP::ZF1::PH(PLC1delta1), unc-119(+)] II; Is[mCherry::HistoneH2B] IV; atg-16.1(gk668615[Q356*]) X*	Crossed VC40503 to WEH95
WEH684	*atg-16.2(ok3224) xnIs390[pie-1::GFP::ZF1::PH(PLC1delta1), unc-119(+)] II; Is[mCherry::HistoneH2B] IV*	Crossed RB2372 to WEH95
WEH696	*Strain lost. atg-16.2(ok3224) xnIs390[pie-1::GFP::ZF1::PH(PLC1delta1), unc-119(+)] / mIn1[dpy-10(e128) mIs14(myo-2::gfp; pes-10::gfp)] xnIs25[cdc-42::GFP::CDC-42; unc-119(+)] II; unc-119(ed3) III; Is[mCherry::HistoneH2B] IV; atg-16.1(gk668615[Q356*]) X*	Crossed WEH683 to WEH684, then to FT97
WEH700	*wurIs144[pGF13: pie-1::ZF1::mCherry::his-15; unc-119(+)] I; unc-119(ed3) ltIs38[pie-1::GFP::PH(PLC1delta1), unc-119(+)] III*	Crossed WEH464 to WEH399
WEH707	*wurIs144[pGF13: pie-1::ZF1::mCherry::his-15; unc-119(+)] I; unc-119(ed3) ltIs38[pie-1::GFP::PH(PLC1delta1), unc-119(+)] III; atg-16.1(gk668615[Q356*]) X*	Crossed WEH683 to WEH700
WEH708	*wurIs144[pGF13: pie-1::ZF1::mCherry::his-15; unc-119(+)] I; atg-16.2(gk145022[W253*]) II; unc-119(ed3) ltIs38[pie-1::GFP::PH(PLC1delta1), unc-119(+)] III*	Crossed VC20426 to N2, then to WEH700
WEH709	*wurIs144[pGF13: pie-1::ZF1::mCherry::his-15; unc-119(+)] I; mIn1[dpy-10(e128) mIs14(myo-2::gfp; pes-10::gfp)] II; unc-119(ed3) ltIs38[pie-1::GFP::PH(PLC1delta1), unc-119(+)] III*	Crossed WEH82 to WEH700
WEH711	*wurIs144[pGF13: pie-1::ZF1::mCherry::his-15; unc-119(+)] I; atg-16.2(gk145022[W253*]) II; unc-*	Crossed WEH707 to WEH708

	119(ed3) ltIs38[pie-1::GFP::PH(PLC1delta1), unc-119(+)] III; atg-16.1(gk668615[Q356]) X*	
WEH714	*wurIs144[pGF13: pie-1::ZF1::mCherry::his-15; unc-119(+)] I; atg-16.2(gk145022[W253*]) II; unc-119(ed3) ltIs38[pie-1::GFP::PH(PLC1delta1), unc-119(+)] III; atg-16.1(gk668615[Q356*]) X*	Crossed WEH708 to WEH707 to WEH709
WEH718	*Si[pVIG57: Pmex-5::mCherry::LGG-2::tbb-2 3'UTR, C.b. unc-119(+)] atg-16.2(gk145022[W253*]) II; unc-119(ed3) ltIs38[pie-1::GFP::PH(PLC1delta1), unc-119(+)] III*	Crossed WEH708 to WEH224
WEH722	*Si[pVIG57: Pmex-5::mCherry::LGG-2::tbb-2 3'UTR, C.b. unc-119(+)] II; unc-119(ed3) ruIs32[pAZ132: pie-1::GFP::H2B, unc-119(+)] III*	Crossed WEH224 to N2
WEH728	*Si[pVIG57: Pmex-5::mCherry::LGG-2::tbb-2 3'UTR, C.b. unc-119(+)] atg-16.2(gk145022[W253*]) II; unc-119(ed3) ruIs32[pAZ132: pie-1::GFP::H2B, unc-119(+)] III*	Crossed WEH722 to WEH718
WEH729	*Si[pVIG57: Pmex-5::mCherry::LGG-2::tbb-2 3'UTR, C.b. unc-119(+)] II; unc-119(ed3) ruIs32[pAZ132: pie-1::GFP::H2B, unc-119(+)] III; atg-9(bp564) V*	Crossed HZ1687 to WEH722
WEH731	*wurIs144[pGF13: pie-1::ZF1::mCherry::his-15; unc-119(+)] I; unc-119(ed3) ltIs38[pie-1::GFP::PH(PLC1delta1), unc-119(+)] III; atg-9(bp564) V*	Crossed HZ1687 to WEH700
WEH734	*Si[pVIG57: Pmex-5::mCherry::LGG-2::tbb-2 3'UTR, C.b. unc-119(+)] II; unc-119(ed3) ruIs32[pAZ132: pie-1::GFP::H2B, unc-119(+)] III; atg-16.1(gk668615[Q356*]) X*	Crossed WEH683 to WEH722
WEH739	*Si[pVIG57: Pmex-5::mCherry::LGG-2::tbb-2 3'UTR, C.b. unc-119(+)] atg-16.2(gk145022[W253*]) II; unc-119(ed3) ruIs32[pAZ132: pie-1::GFP::H2B, unc-119(+)] III; atg-16.1(gk668615[Q356*]) X*	Crossed WEH734 to WEH728
WEH751	*wurSi2[pVIG57-CTPD: Pmex-5::CTPD::mCherry::LGG-2::tbb-2 3'UTR, C.b. unc-119(+)] II; unc-119(ed3) III*	Homozygosed from worms injected by InVivo Biosystems
WEH755	*wurSi2[pVIG57-CTPD: Pmex-5::CTPD::mCherry::LGG-2::tbb-2 3'UTR, C.b. unc-119(+)] II; unc-119(ed3) ruIs32[pAZ132: pie-1::GFP::H2B, unc-119(+)] III*	Crossed AZ212 to WEH751

References

38. Praitis V, Casey E, Collar D, Austin J. Creation of low-copy integrated transgenic lines in Caenorhabditis elegans. Genetics. 2001;157(3):1217-26.

39. Lin L, Yang P, Huang X, Zhang H, Lu Q, Zhang H. The scaffold protein EPG-7 links cargo–receptor complexes with the autophagic assembly machinery. Journal of Cell Biology. 2013;201(1):113-29.

40. Montoyo-Rosario JG, Armenti ST, Zilberman Y, Nance J. The role of pkc-3 and genetic suppressors in Caenorhabditis elegans Epithelial Cell Junction Formation. Genetics. 2020;214(4):941-59.

41. Olson SK, Bishop JR, Yates JR, Oegema K, Esko JD. Identification of novel chondroitin proteoglycans in Caenorhabditis elegans: embryonic cell division depends on CPG-1 and CPG-2. The Journal of cell biology. 2006;173(6):985-94.

42. Consortium CeDM. Large-scale screening for targeted knockouts in the Caenorhabditis elegans genome. G3: Genes| Genomes| Genetics. 2012;2(11):1415-25.

43. Alberti A, Michelet X, Djeddi A, Legouis R. The autophagosomal protein LGG-2 acts synergistically with LGG-1 in dauer formation and longevity in C. elegans. Autophagy. 2010;6(5):622-33.

44. Beer KB, Rivas-Castillo J, Kuhn K, Fazeli G, Karmann B, Nance JF, et al. Extracellular vesicle budding is inhibited by redundant regulators of TAT-5 flippase localization and phospholipid asymmetry. Proceedings of the National Academy of Sciences. 2018;115(6):E1127-E36.
