# Supplementary material for: Phagolysosomes break down the membrane of a non-apoptotic corpse independent of macroautophagy
Source: PLoS One. 2024 Nov 21;19(11):e0306435. doi: 10.1371/journal.pone.0306435 (PMC11581207; doi:10.1371/journal.pone.0306435)
Supplement: S2 Table — (PDF) [file pone.0306435.s005.pdf]

S2 Table. Oligonucleotide primers.

**Primer**	**Sequence**	**Genotyped Allele or Purpose**
atg-9 exon 5 F2	CTGGATCACTCGGGTCATCAAA	*atg-9(bp564)*
atg-9 intron 5 R	CCGAATTTCAGGCGATTTTCAGACA	*atg-9(bp564)*
gk668615 HinfI F	GTTATCAAAATTTGGGAgAT	*atg-16.1(gk668615)*
atg-16.1 exon 10R	GATCCGTTGACCTTCCATCGT	*atg-16.1(gk668615)*
atg-16.2 exon 1 F	GGCTGACAGTGAATCTCGTT	*atg-16.2(ok3224),* RT-PCR
ok3224 InnerLeft	CCGTATGGCCAGAAAACGTA	*atg-16.2(ok3224)*
atg-16.2 exon 2 R	CATCTGTGCGCGTTCTTCTT	*atg-16.2(ok3224),* RT-PCR
atg-16.2 exon 2 F3	ACGCAGCTAGATAACGAACGAT	*atg-16.2(gk145022)*
gk145022 HinfI R	GCTGTCGCAAATGTATCATCAGAGA	*atg-16.2(gk145022)*
oJN27	GTCCTTCTTGAGTTTGTAACAGC	*ltIs38*
ZF1 NheI R	CCTGCTAGCCCTCGGAACTCTCAGCTCAT	*ltIs38*
ttTi5605 F2	TGCTTATCTCGAATGAGACCCTT	*Si[pVIG57]*
ttTi5605 R	GACACCCGGGTTTGTCTAGAT	*Si[pVIG57]*
Cb-unc-119N R	GAGCTGGGGAGAAGAAGACACT	*Si[pVIG57]*
tat-5 geno F	TGCTCCAATCACTTACTGGGGAC	RT-PCR
tat-5 geno R	TACGCGGAGTGAAATTGGAATAA	RT-PCR
mex-5p oma-1(219) F	CTCATTGTATTCTCTCTTAATTAATTTTATCGATAATCAATTGAATGTTTCAGACAGAGAATGATTGCCGCTCCCCCACTTTCTG	CTPD subcloning
mCh oma-1(378) R	TTATCTTCTTCACCCTTTGAGACATCAGTTGCGACAGATTTCATCAGAAGAGATTGAGCA	CTPD subcloning
